# Supplementary material for: Analysis of gut microbiota in patients with AVS and identification of potential biomarkers: a cross-sectional study
Source: Microbiol Spectr. 2025 Oct 27;13(12):e03215-24. doi: 10.1128/spectrum.03215-24 (PMC12671120; doi:10.1128/spectrum.03215-24)
Supplement: Supplemental data — Supplementary statistical analyses. [file spectrum.03215-24-s0001.docx]

**Supplementary statistical analyses**

**Statistical analyses**

Statistical analyses were conducted using IBM SPSS Statistics for Windows 26.0 IBM (Corp., Armonk, N.Y., USA) and R version 3.5.1 (R Foundation for Statistical Computing, Vienna, Austria). The Shapiro-Wilk test was deployed to determine if the continuous variables conform to a normal distribution. Normally distributed data are presented as mean ± SD, and Student t-tests were employed to conduct comparisons between groups. For abnormally distributed variables, data were presented as median (interquartile range), with comparisons conducted utilizing the nonparametric Wilcoxon test. Categorical data were presented as frequencies and percentages, with comparisons made using the χ² test or Fisher’s exact test, as appropriate. Random forest models were trained using the R random Forest package to predict AVS. A probability matrix generated during the random forest procedure, along with a sample metadata file, was employed to generate the decision curve analysis (DCA) in R. Spearman's rank correlation was utilized to assess the relationships among non-normally distributed data. *P*-values were adjusted for multiple comparisons using the Benjamini-Hochberg method, which controls the false discovery rate, ensuring more robust conclusions from the analyses A *P*-value < 0.05 was considered statistically significant.

**Random Forest**

The random forest (RF) model was employed as an ensemble learning method for microbiome feature selection and classification . RF operates by constructing multiple decision trees during training, where each tree votes on the final prediction, thereby reducing overfitting through majority voting (Breiman, 2001).

**Key parameters included:**

- 500 trees with Gini index as the splitting criterion (mean decrease in impurity ≥0.01)
- Node size of 5 and mtry = √p (where p = number of features)
- “All” strategy: Retaining all ASVs with non-zero importance scores for downstream analysis
- “None” strategy: Excluding ASVs with negative importance scores

**Model Validation**

- 10-fold nested CV: Outer loop (10 folds) for performance estimation; inner loop (5 folds) for hyperparameter tuning
- Leave-one-out CV (LOOCV): Iteratively training on n-1 samples and testing on the held-out sample
- Cutoffs for model acceptance: AUC >0.85 (ROC analysis), accuracy >80% (95% CI), and F1-score >0.75 based on permutation testing (1000 iterations).

The random forest (RF) model was employed as an ensemble learning method for microbiome feature selection and classification (Figure X). RF operates by constructing multiple decision trees during training, where each tree votes on the final prediction, thereby reducing overfitting through majority voting (Breiman, 2001).

**Key parameters included:**

- 500 trees with Gini index as the splitting criterion (mean decrease in impurity ≥0.01)
- Node size of 5 and mtry = √p (where p = number of features)*
- “All” strategy: Retaining all ASVs with non-zero importance scores for downstream analysis
- “None” strategy: Excluding ASVs with negative importance scores

**Model Validation**

- 10-fold nested CV: Outer loop (10 folds) for performance estimation; inner loop (5 folds) for hyperparameter tuning
- Leave-one-out CV (LOOCV): Iteratively training on n-1 samples and testing on the held-out sample
- Cutoffs for model acceptance: AUC >0.85 (ROC analysis), accuracy >80% (95% CI), and F1-score >0.75 based on permutation testing (1000 iterations).

**Construction of the receiver operating characteristic curve (ROC)**

The input samples (the biomarker data) were partitioned into 10 subsets, 8 subsets were used to fit random forest models in the scikit-learn package, and the remaining subset was used to calculate prediction probabilities. This cross-prediction process was then repeated 1000 times using bootstrap sampling to ensure robustness. The prediction results from each bootstrap iteration were used to calculate the AUC and its 95% confidence intervals (CI) using the percentile method (2.5% and 97.5% percentiles). Finally, the ROC curve was plotted with the AUC value and its confidence interval displayed on the graph.

**Decision curve analysis (DCA)**

Two main steps were performed to plot the DCA curve:

Step1: A random forest model was fitted using the biomarker data in the scikit-learn library, and the predicted probabilities for the test set were generated.

Step 2: The predicted probabilities and the true labels were used to calculate the net benefit at various threshold probabilities.

The DCA curve was then plotted using the `plot_DCA` function, which visualizes the net benefit of the model compared to the "treat all" and "treat none" strategies across a range of threshold probabilities.

**Correlation analysis of the genera and the clinical indices**

"Spearman's rank correlation analyses were conducted between microbial features (Twenty-four genera which potentially serve as biomarkers in AVS diagnosis) and clinical variables using the R package stats (v4.2.2). Correlation heatmap generated using ComplexHeatmap (v2.14.0), with dendrograms computed by hclust using complete linkage method. Color scale represents *P* values from -1 (blue) to +1 (red), with asterisks indicating significance levels (*FDR<0.05; **FDR<0.01).

Line charts created with ggplot2 (v3.4.0) using purple dotted line for trend visualization. The analysis included:

- Computation of correlation coefficients (R) with significance testing via asymptotic t approximation
- Adjustment for multiple comparisons using Benjamini-Hochberg false discovery rate (FDR) correction
- Retention of correlations meeting FDR <0.05 and |R| >0.3 thresholds
- The resulting correlation matrix was visualized through Hierarchical clustering heatmaps
