# Supplementary material for: Analysis of gut microbiota in patients with AVS and identification of potential biomarkers: a cross-sectional study
Source: Microbiol Spectr. 2025 Oct 27;13(12):e03215-24. doi: 10.1128/spectrum.03215-24 (PMC12671120; doi:10.1128/spectrum.03215-24)
Supplement: Supplemental material — s and methods description of 16SrDNA sequencing. [file spectrum.03215-24-s0002.docx]

**Material and method description of 16SrDNA sequencing**

**1. Experimental process**

**1.1 Extraction of total DNA from microbiome**

Total fecal microbial DNA was extracted using the Fecal Genome DNA Extraction Kit (AU46111-96, BioTeke, China) by LC-Bio, following ISO 9001-certified protocols in strict adherence to the manufacturer’s instructions. The DNA was quantified by Qubit (Invitrogen, USA). The process was strictly performed according to the manufacturer's protocol.Total DNA was amplified by PCR using the universal primer 341F/805R(341F: 5’-CCTACGGGNGGCWGCAG-3’; 805R:5’-GACTACHVGGG TATCTAATCC-3’).

**1.2 PCR Amplification**

Table 1 PCR reaction system

| **PCR reaction component PCR reaction volume** | |
| --- | --- |
| Phusion Hot start flex 2X Master Mix | 12.5μL |
| Forward Primer | 2.5μL |
| Reverse Primer | 2.5μL |
| Template DNA | 50 ng |
| ddH_2_O | 25μL |

PCR reaction conditions: According to the reaction system, PCR reagent was added for PCR amplification and pre-denatured at 98℃ for 30s. Denatured at 98℃ for 10 seconds, annealed at 54℃ for 30 seconds, extended at 72℃ for 45 seconds, 32 cycles; Finally, it is extended for 10 minutes at 72℃.

Since ultrapure water, rather than sample solution, is used throughout the DNA extraction process, the possibility of a false-positive PCR result acting as a negative control can be ruled out.

**1.3 PCR product quantification**

The PCR product was purifed using AMPure XP Beads (Beckman Coulter Genomics, Danvers, MA, USA) and quantifed using Qubit (Invitrogen, USA).

**1.4 Library mixed samples on machine sequencing**

The purified PCR products were evaluated using the Agilent 2100 Bioanalyzer (Agilent, USA) and the library quantification kit from Illumina (Kapa Biosciences, Woburn, MA, USA). Qualified library concentration should be above 2nM. The qualified sequencing libraries (with non-repeating index sequences) were serially diluted and mixed in the appropriate proportions according to the required sequencing amount. The libraries were then denatured into single-stranded DNA using NaOH before being loaded for sequencing. Sequencing was performed using the NovaSeq 6000 platform with 2×250 bp paired-end sequencing, and the corresponding reagent was the NovaSeq 6000 SP Reagent Kit (500 cycles).

1. **Data analysis**

2.1 Data Splitting

The paired-end sequencing data obtained must be split based on barcode information, and adapter and barcode sequences should be removed.

2.2 Data Assembly and Filtering

1) Remove primer sequences and adapter sequences from the RawData. (Software: cutadapt (v1.9), Parameters: '-g R1 -G R2 -n 1 -O 17 -m 100').

2) Merge each pair of paired-end reads based on the overlap region into a longer tag. (Software: FLASH (v1.2.8), Parameters: '-m 10 -M 100 -x 0.25 -t 1 -z').

3) Perform quality scanning of the sequencing reads using a sliding window method. The default window size is 100 bp, and when the average quality value within the window is lower than 20, the read is trimmed from the starting point of the window to the 3' end. (Software: fqtrim, Parameters: '-P 33 -w 100 -q 20 -l 100 -m 5 -p 1 -V -o trim.fastq.gz').

4) Remove sequences shorter than 100 bp after trimming.

5) Remove sequences where the content of N (uncertain ambiguous bases) exceeds 5%.

6) Remove chimeric sequences. (Software: Vsearch (v2.3.4), Parameters: default).

**2.3 DADA2 Denoising**

Perform length filtering and denoising using DADA2 through the command qiime dada2 denoise-paired. This process generates ASV feature sequences and an ASV abundance table, while removing singletons ASVs (i.e., ASVs that appear only once across all samples, which is the default action), and ASVs less than 0.001% were removed.

**2.4 Diversity Analysis**

**Alpha Diversity measures and calculations**

Alpha diversity analyses are performed based on the obtained ASV feature sequences and ASV abundance tables. The Chao 1 and Shanon indices were used to analyze Alpha diversity, were calculated using the vegan package 2.5-5.

**Beta Diversity measures and comparisons**

Beta-diversity analyses are performed based on the obtained ASV feature sequences and ASV abundance tables. The unweighted_unifrac dissimilarity , a commonly used Beta-diversity index, was used in our study, The unweighted_unifrac dissimilarity between the two groups was compared by the adonis in the vegan package. and a principal coordinate analysis (PCoA) analysis was performed using QIIME based on the unweighted_unifrac distance. Beta-diversity analyses were performed based on the unweighted_unifrac distance, and Adonis was used to estimate the amount of dissimilarity in microbial compositions between groups .

**2.5 Species Annotation**

Species annotation is performed based on the ASV sequence file using the SILVA database (Release 138, https://www.arb-silva.de/documentation/release138/, annotation threshold: --min_confidence 0.7) and the NT-16S database (Release 20230718, annotation threshold: --min_ident 90 --min_cov 80 --max_e 1e-5). The abundance of species at each taxonomic level in the samples is then calculated based on the ASV abundance table.

**2.6 Differential Analysis**

Differential analysis is performed between comparison groups based on the species abundance statistics. Different statistical methods are chosen depending on the sample type: Fisher's exact test is used for comparisons of samples without biological replicates; Mann-Whitney U test (also known as the Wilcoxon rank-sum test) is used for comparisons between two groups with biological replicates. The linear discriminant analysis effect size (LEfSe) method was used to identify features that differed between the groups. The threshold of the logarithmic LDA score for discriminative features was set to 3.0. The significance threshold was set to *P* < 0.05.

**2.7 Phylogenetic Analysis**

The phylogenetic tree was constructed based on the ASV representative sequences using QIIME2's align-to-tree-mafft-fasttree pipeline (QIIME2 version 2023.5). Briefly, sequences were aligned with MAFFT (v7.505) using the default parameters, followed by masking of highly variable positions to reduce noise. An unrooted tree was then generated with FastTree (v2.1.11) under the GTR+CAT model with 1000 bootstrap replicates to assess branch support. Finally, the tree was rooted at midpoint using the qiime phylogeny midpoint-root command for downstream analyses.

1. **Note**

**Environmental and Laboratory Contaminants:** Strict protocols were followed to minimize environmental contamination during sample collection, processing, and sequencing. These protocols included the use of sterile equipment, conducting procedures in a controlled environment, and regular monitoring of laboratory conditions. DNA extraction and PCR amplification were carried out according to standardized protocols, with automated extraction to reduce human-handling contamination.

**Tag Jumping and Cross-Contamination:** We implemented stringent quality control measures, including the use of unique barcodes for each sample and proper PCR clean-up procedures, to prevent tag jumping and cross-contamination during sequencing. Additionally, the laboratory conducts regular environmental monitoring, and extraction and amplification are performed in separate areas to further minimize the risk of cross-contamination.
